# Quadriceps and Patellar Tendon Thickness and Stiffness in Elite Track Cyclists: An Ultrasonographic and Myotonometric Evaluation

**DOI:** 10.3389/fphys.2020.607208

**Published:** 2020-12-14

**Authors:** Sebastian Klich, Krzysztof Ficek, Igor Krymski, Andrzej Klimek, Adam Kawczyński, Pascal Madeleine, Cesar Fernández-de-las-Peñas

**Affiliations:** ^1^Department of Paralympic Sport, University School of Physical Education in Wrocław, Wrocław, Poland; ^2^Department of Physiotherapy, Academy of Physical Education in Katowice, Katowice, Poland; ^3^Galen Orthopedic Center, Bieruń, Poland; ^4^Polish Cycling Federation, Pruszków, Poland; ^5^Biomedical Science Institute, University School of Physical Education in Kraków, Kraków, Poland; ^6^Department of Health Science and Technology, Sport Sciences—Performance and Technology, Aalborg University, Aalborg, Denmark; ^7^Department of Physical Therapy, Occupational Therapy, Rehabilitation, and Physical Medicine, Universidad Rey Juan Carlos, Alcorcón, Spain; ^8^Cátedra Institucional en Docencia, Clínica e Investigación en Fisioterapia: Terapia Manual, Punción Seca y Ejercicio Terapéutico, Universidad Rey Juan Carlos, Alcorcón, Spain

**Keywords:** tendon, thickness, stiffness, maps, track cycling

## Abstract

Track cyclists are required to perform short- and long-term efforts during sprint and endurance race events, respectively. The 200 m flying sprint races require maximal power output and anaerobic capacity, while the 4,000 m pursuit cyclists demand a high level of aerobic capacity. Our goal was to investigate spatial changes in morphological and mechanical properties displayed using 3D topographical maps of the quadriceps muscle and tendons after 200 m flying start and 4,000 m individual pursuit race in elite track cyclists. We hypothesized a non-uniform distribution of the changes in the quadriceps muscle stiffness (QM_stiff_), and acute alterations in quadriceps tendon (QT_thick_) and patellar tendon (PT_thick_) thickness. Fifteen men elite sprint and 15 elite men endurance track cyclists participated. Sprint track cyclists participated in a 200 m flying start, while endurance track cyclists in 4,000 m individual pursuit. Outcomes including QT_thick_ (5–10–15 mm proximal to the upper edge of the patella), PT_thick_ (5–10–15–20 mm inferior to the apex of the patella)—using ultrasonography evaluation, QM_stiff_, and quadriceps tendon stiffness (QT_stiff_) were measured according to anatomically defined locations (point 1–8) and patellar tendon stiffness (PT_stiff_)—using myotonometry, measured in a midway point between the patella distal and the tuberosity of tibial. All parameters were assessed before and after (up to 5 min) the 200 m or 4,000 m events. Sprint track cyclists had significantly larger QT_thick_ and PT_thick_ than endurance track cyclists. *Post-hoc* analysis showed significant spatial differences in QM_stiff_ between rectus femoris, vastus lateralis, and vastus medialis in sprint track cyclists. At before race, sprint track cyclists presented significantly higher mean QT_thick_ and PT_thick_, and higher QM_stiff_ and the QT_stiff_, as compared with the endurance track cyclists. The observed changes in PT_Thick_ and QT_Thick_ were mostly related to adaptation-based vascularity and hypertrophy processes. The current study suggests that assessments using both ultrasonography and myotonometry provides crucial information about tendons and muscles properties and their acute adaptation to exercise. Higher stiffness in sprint compared with endurance track cyclists at baseline seems to highlight alterations in mechanical properties of the tendon and muscle that could lead to overuse injuries.

## Introduction

Track cycling can be divided into two main categories: sprint (≤ 1,000 m) and endurance (>1,000 m) events. The most popular individual races, in those categories, are 200 m flying start and 4,000 m pursuit, that last on average 10 s and >4 min, respectively (Craig and Norton, 2001). Sprint and endurance track cyclists have different anthropometric characteristics, muscle's mechanical properties, and fiber type composition. For instance, sprinters exhibit greater thigh girths but shorter thigh lengths (Van Der Zwaard et al., 2019) and their quadriceps muscle contains predominantly fast-twitch fibers (Loturco et al., 2015). Cyclists are more exposed to tendon injuries (i.e., patellar quadriceps tendinopathies) than muscle sprains (Wanich et al., 2007). Further, sprint track cyclists have also a higher risk of tendinopathy than endurance cyclists (Craig and Norton, 2001; Klich et al., 2020). Penailillo et al. (2015) showed that 25% of cyclists suffer from patellar tendon (PT) pain. Quadriceps tendon (QT) pain also appears frequently in cyclists, particularly in sprinters, as a result of overuse syndrome on the lateral side of the knee (Wanich et al., 2007).

Quadriceps and patellar tendinopathy should be diagnosed by noninvasive, real-time methods based on measures of thickness and mechanical properties of PT and QT tendons (Loturco et al., 2015; Klich et al., 2018, 2020). Mechanical loadings within thigh muscles can affect QT and PT thickness as well as stiffness (Loturco et al., 2015; Klich et al., 2018). Thus, the assessment of thigh muscles and tendons using ultrasonography and myotonometry could be used to assess the effects of muscle loading during cycling competitions. Diagnostic ultrasound imaging and myotonometry have been proposed as reliable research and clinical instruments to evaluate morphological and mechanical properties of skeletal muscles (Bizzini and Mannion, 2003; Klich et al., 2019). Diagnostic ultrasound imaging has been previously used to assess the reliability and magnitude of QT and PT characteristics in volleyball players (Kulig et al., 2013; Visnes et al., 2015). Kulig et al. (2013) demonstrated an increase in proximal PT thickness (PT_Thick_) in symptomatic volleyball players when compared with those without symptoms. Visnes et al. (2015) have reported a relationship between QT and PT thickness and symptoms of jumper's knee (a common syndrome related to morphological alterations in tendon thickness and increased vascularity).

Myotonometry has been also used to assess the stiffness of quadriceps muscle (QM_stiff_) and PT (PT_stiff_) in individual and team sport athletes (Young et al., 2018; Chen et al., 2019; Klich et al., 2020) and also after acute injury (Liang et al., 2017). Young et al. (2018) have shown higher PT_stiff_ in break-dancers compared with a control group, as a sign of adaptation after training. Klich et al. (2020) found an increase in QM_stiff_ after 200 m flying start and sprints in a case study. Among soccer players compared with healthy sedentary participants, both increased rectus femoris and lower PT and QT stiffness have been recently reported (Taş et al., 2019). Chino et al. (2018) showed lower stiffness of rectus femoris muscle in athletes, compared with non-athletes. The application of those ultrasonic and myotonometric measurements may help detect acute changes following flying sprint or sprint race.

Field investigations are needed in professional sport to assess changes in muscle and tendon load in real-time during training and competitions (Elliott and Alderson, 2007). Moreover, ultrasound imaging and myotonometry are needed to indicate the development of soft tissue thickness and stiffness. To the best of our knowledge, no study has assessed tendon thickness and stiffness of QT and PT before and after cycling events in the elite sprint and endurance track cyclists. These outcomes may provide a better understanding of QT and PT changes that could help to understand better tendinopathy mechanisms. Thus, the current study was designed to quantify changes in QT_thick_ and PT_thick_ as well as changes in QM_stiff_, QT_stiff_, and PT_stiff_ at several locations of the dominant lower limb before and after sprint and endurance race competition on 200 m flying start and 4,000 m individual pursuit, respectively. Furthermore, to ensure that such assessments are reliable, we also reported the test-retest reliability of tendons thickness and stiffness among elite track cyclists. Therefore, we aimed to investigate changes in morphological and mechanical properties in a real-time competition of elite track cyclists. The secondary aim was to assess cross-sectional comparisons between sprint and endurance cyclists at baseline. We have hypothesized a non-uniform distribution of the changes in the QM_stiff_, and acute alterations in QT_thick_ and PT_thick_, in both sprint and endurance track cyclists.

## Materials and Methods

### Participants

We tested a group of 15 sprint track cyclists (all men, mean ± SD age 26 ± 4 years, body height 183 ± 5.4 cm; body mass 88 ± 4.5 kg; BMI: 25.3 ± 0.7 kg/m^2^; thigh length 54.4 ± 2.8 cm) and 15 endurance track cyclists (all men, mean age 23 ± 1.8 years, body height 191 ± 3.6 cm; body mass 76 ± 3.5 kg; BMI: 21.1 ± 0.7 kg/m^2^; thigh length 61.1 ± 1.7 cm). The thigh length was defined as distance from the anterior superior iliac spine to medial joint line (Goyal et al., 2020). All subjects were tested to identify the dominant leg during pedaling (Watanabe et al., 2016). All (*n* = 30) subjects responded that they used their right leg during the down-stroke phase of the pedaling cycle. All measurements were conducted on the dominant side (right side). The track cyclists were competitors of the Polish national team specialized in either sprint or endurance events with a mean training experience of 11.5 ± 1.4 years. Twenty-two participants (73%) were professional track cyclists, including four winners of world cups. Participants were competing at the international-level track races (World Cup, European Championship, and World Championship). The exclusion criteria for both groups included: (1) current or previous thigh and knee injury or symptoms; and (2) prior history of surgery in the lower extremity.

### Study Design

This study cross-sectional used a repeated-measures design performed during sports competition. The assessments were made *in situ*, on a velodrome. Sprint track cyclists participated in 200 m flying start, while endurance track cyclists in 4,000 m individual pursuit. The test-retest relative and absolute reliability was also investigated for thickness and stiffness measurements collected twice before the races. The thickness (PT_thick_ and QT_thick_) and stiffness of the (QM, QT, and PT) were measured at several locations before and after (up to 5 min) 200 and 4,000 m races, respectively. The total time of preparing all measurements took ~1–1.5 min per each participant (including myotonometry, e.g., 30 s and US, e.g., 30 s −1 min). The study was conducted following STROBE guidelines (Von Elm et al., 2008).

### Ultrasound Assessment

Ultrasonography was performed using an ultrasound scanner (HS-2200, Honda, Toyohashi, Japan) with a 7.5 (6.0–11.0) MHz and 40 mm linear array transducer (HLS-584 M, Honda, Toyohashi, Japan) in gray scale B-mode. The settings of the ultrasound system were standardized for all participants and kept identical for all measures. The scan depth was set to 1.8 mm, in agreement with Skou and Aalkjaer (2013). The axial resolution of ultrasound images was found to be 0.068 mm per pixel. Measurements of PT_thick_ and QT_thick_ were performed according to recommendations of the European Society of Musculoskeletal Radiology (Beggs et al., 2016).

**Figure 1:**
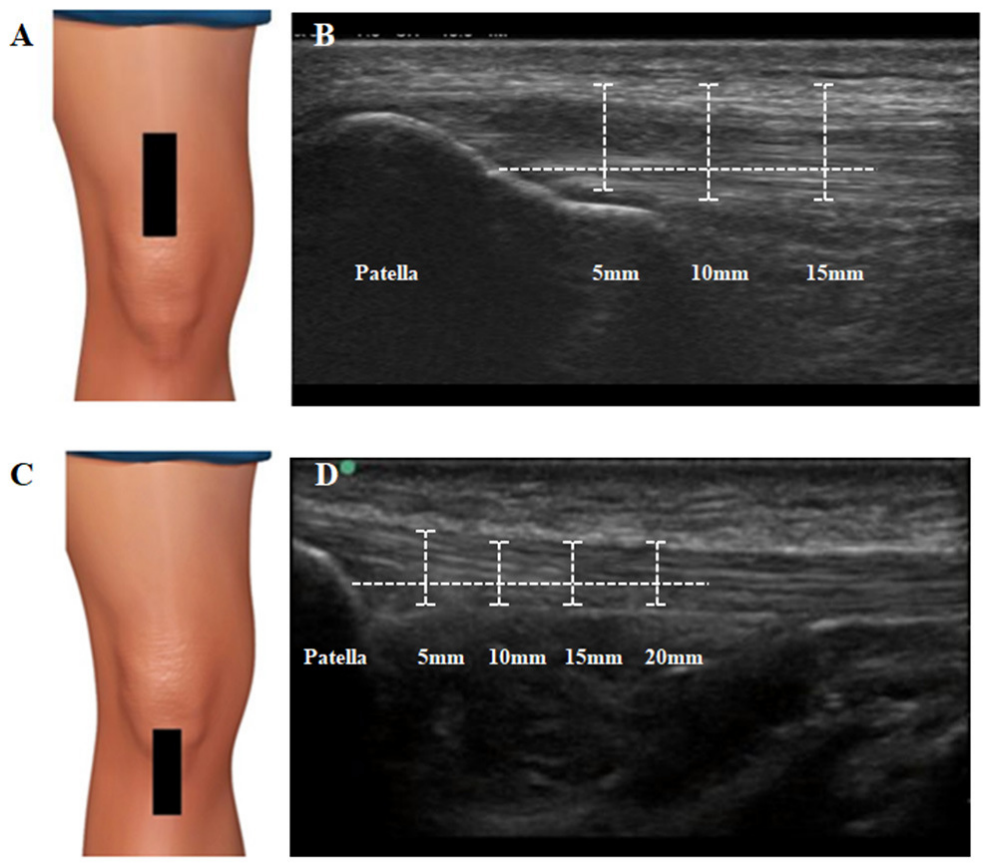
Ultrasound assessment and measurement of quadriceps and patellar tendon. **(A)** Transducer placement for quadriceps tendon. The transducer was placed in the long axis of this tendon, proximal to the upper edge of the patella. **(B)** Measurement procedures for quadriceps tendon thickness. Thickness was assessed on three points along the QT located at 5–10–15 mm lateral to the reference point (hyperechoic region of the patella). The QT borders were defined inferiorly as the first hyperechoic region between superficial and deep layers. **(C)** Transducer placement for patellar tendon. The linear transducer was placed longitudinally distal to the patella. **(D)** Measurement procedures for patellar tendon thickness. Thickness was assessed on four locations, set at 5–10–15–20 mm inferior to the apex of the patella. Tendon borders were defined inferiorly as the first hyperechoic region between the subcutaneous tissue and the deep fascia layer.

Participants were lying in a supine position with their right knee (dominant side) flexed at ~30° (Giombini et al., 2013). A pillow was placed under the popliteal space during the assessments. This knee position avoids possible anisotropy related to the concave profile as a result of posterior thigh muscles and PT extension (Skou and Aalkjaer, 2013). For QT, the transducer was placed in the long axis of this tendon, proximal to the upper edge of the patella. Thickness was assessed on three points along the QT located at 5–10–15 mm lateral to the reference point (hyperechoic region of the patella). The QT borders were defined inferiorly as the first hyperechoic region between superficial and deep layers. The three measures (5–10–15 mm) were averaged for a single measure of tendon thickness. For PT, the linear transducer was placed longitudinally distal to the patella. The thickness of the PT was assessed on four locations, set at 5–10–15–20 mm inferior to the apex of the patella. Tendon borders were defined inferiorly as the first hyperechoic region between the subcutaneous tissue and the deep fascia layer. The four measures (5–10–15–20 mm) were averaged for a single measure of tendon thickness (Figure 1).

The data collection for reliability assessment took place 2 days before the main procedure. Sprint and endurance track cyclists were asked to avoid physical effort and had none training session before the reliability data collection. A single examiner (SK) took two US images for each tendon (QT and PT). To avoid a learning effect, all subjects were coded by assigning an individual ID number. The data measurements for QT and PT thickness were performed 1 week later. The order in which the images were assessed was randomized, however, the examiner was blinded to each measurements and the group (sprint and endurance) to which the subject was assigned. The measured QT and PT thickness were averaged and used for further statistical analysis.

During the experimental protocol two images were taken for each tendon, and were averaged for data analysis. The topographical maps of the PT and QT were generated using Matlab (version R2017b, The Mathworks, Natick MA, United States of America) by means of an inverse distance weighted interpolation of the averaged values of each location (5–10–15 mm and 5–10–15–20 mm) to obtain a 3D graphical representation (Binderup et al., 2010; Fernández-Carnero et al., 2010) of the changes in tendon thickness. Of note, the 3D graphical representation are only used for visualization purposes.

### Myotonometric Assessment

A hand-held myotonometer device (MyotonPro, Myoton Ltd., Estonia) was used to measure QM_stiff_ (including QT_stiff_) and PT_stiff_ at several locations. Muscle stiffness is defined as the property that characterizes resistance to the contraction of the external stretching force that deforms the initial shape of the tissue. Stiffness (N/m) was computed as *S* = *a*_max_**.***m*_probe_/Δ*l*, where *a* is the acceleration of the damped oscillation; *m*_*probe*_ is the mass of the measurement mechanism and Δ*l* is the probe displacement (Klich et al., 2019). The examiner located the probe perpendicular to the tested area. Then, the probe generated three impulses exerted on the testing area.

**Figure 2:**
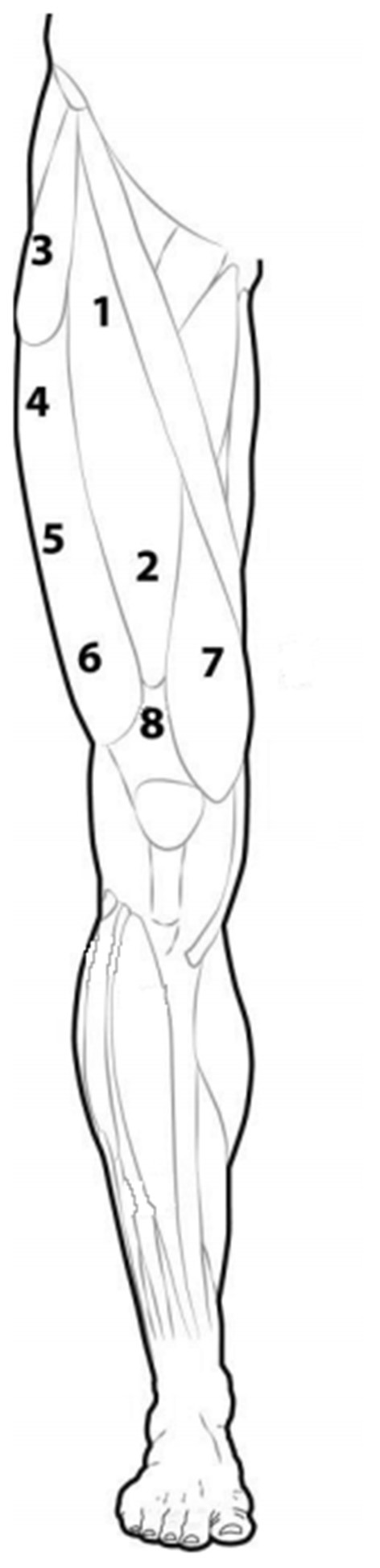
Schematic representation of the eight points for the quadriceps muscle and tendon stiffness measure.

Participants were lying in a supine position with feet on the massage table. The QM_stiff_ measurements were made on the dominant lower extremity over eight reference points, including rectus femoris (RF) (no. 1–2); tensor fasciae latae (TFL) (no. 3); vastus lateralis (VL) (no. 4-6), vastus medialis (VM) (no. 7) and quadriceps tendon (QT) (no. 8) in line with previous studies (Domínguez-Martín et al., 2013; Kawczynski et al., 2014). The reference points for RF were musculotendinous points located distal from the initial attachment (no. 1) and terminal attachment (no. 2); TFL was placed in line between anterior superior iliac spine and greater trochanter; VL includes three points on the muscle belly in three equal parts; VM was marked in the middle of the muscle belly. The QT_stiff_ (no. 8) was assessed in the 1/3 proximal between the upper edge of patella and point 2 (RF) (Figure 2), while PT_stiff_ was measured at a midway point between the patella distal and the tuberosity of tibial when the knee was flexed at 90° (Chen et al., 2019).

The data collection for reliability assessment took place 2 days before the main procedure. Sprint and endurance track cyclists were asked to avoid physical effort and had none training session before the reliability data collection. A single examiner (IK) took the measurement of muscle stiffness. The stiffness values of a each point of the quadriceps muscle were measured twice. The data was used to assess absolute and relative reliability for each group of cyclists.

Stiffness measurements were also used to obtain 3D graphical representation of the spatial distribution of the stiffness (Domínguez-Martín et al., 2013; Kawczynski et al., 2014). Similar to the thickness maps, the 3D graphical representation of stiffness were only used for visualization purposes.

### Statistical Analysis

The G^*^Power software (version 3.1.9.2; Kiel University, Kiel, Germany) (Faul et al., 2007) was used to estimate the required sample size setting a minimum expected effect size (Cohen's *f* ) of 0.6, an α level of 0.05, and a power (1–β) of 0.8. The procedure returned a minimum number of 12 participants per group.

The SPSS 18 statistical software (SPSS Inc., Chicago, Illinois, USA) was used for data analysis. Mean values ± standard deviation (SD) are reported. Group differences (track and endurance track cyclists) in anthropometric characteristics were assessed using the independent *t*-test. The relative reliability was calculated using intra-class correlation coefficients (ICCs) using a two-way random effect, absolute agreement (ICC_2, 1_) for both groups. Reliability was classified as poor (ICC < 0.50), moderate (0.50 < ICC < 0.69), good (0.70 < ICC < 0.89) or excellent (ICC ≥ 0.90) (Landis and Koch, 1977). The absolute reliability was evaluated by computing the standard error of measurement (SEM) and the minimal detectable change (MDC) (Weir, 2005).

Two-way analysis of variance with repeated measures (RM-ANOVA) with *time* (pre-post) and *location* (5–10–15 mm for QT_thick_, 5–10–15–20 mm for PT_thick_, 1–2–3–4–5–6–7–8 for QM_stiff_ and PT_stiff_) as within-group factors were conducted for each group. *Post-hoc* tests with Bonferroni corrections were applied when needed. Moreover, a one-way ANOVA was used to assess between-group differences in tendons thickness and stiffness at baseline, as well as, in relative changes in tendons thickness and stiffness [i.e., difference (Δ) between post- and pre-values]. *Post-hoc* tests with Bonferroni corrections were also applied. A *p* ≤ 0.05 was considered as statistically significant. Effect size was estimated using partial eta square (η^2^), classified as small (0.20 < η^2^ < 0.49), medium (0.50 < η^2^ < 0.79), or large (η^2^ >≥ 0.80) (Richardson, 2011).

## Results

### Anthropometric Characteristics

Subjects were not statistically different with respect to age (*p* = 0.07). Sprint track cyclists had significantly higher body mass [*t*_(15)_ = 3.8; *p* = 0.002] and BMI [*t*_(15)_ = 28.2; *p* = 0.003]. On the contrary, endurance track cyclists had significantly higher body height [*t*_(15)_ = −3.7; *p* = 0.001] and thigh length [*t*_(15)_ = −20.7; *p* = 0.001].

### Reliability

The relative reliability was excellent for QT_thick_ (ICC_2, 1_ 0.91, 95%CI: 0.50–0.94) and good for PT_thick_ (ICC_2, 1_ 0.87, 95%CI: 0.35–0.95) in sprint track cyclists. Likewise, the relative reliability was excellent for QT_thick_ (ICC_2, 1_ 0.90, 95%CI: 0.55–0.92) and good for PT_thick_ (ICC_2, 1_ 0.84, 95%CI: 0.40–0.91) in endurance track cyclists. The absolute reliability showed that SEMs and MDCs were lower among the sprinters than among endurance track cyclists. For QT_thick_ and PT_thick_, the SEMs were 0.04 and 0.02 mm in sprint track cyclists, while 0.08 and 0.06 mm in endurance track cyclists. The MDCs were 1.1 and 0.7 mm in sprinters, while 1.5 and 1.0 mm in endurance track cyclists. Moreover, the relative reliability was excellent for stiffness in sprint (ICC_2, 1_ 0.92, 95%CI: 0.50–0.97) and endurance track cyclists (ICC_2, 1_ 0.94, 95%CI: 0.55–0.95). The SEMs and MDCs were 18.20 and 48.16 N/m in sprinters and 17.8 and 45.4 N/m in endurance track cyclists.

### Ultrasound

**Figure 3:**
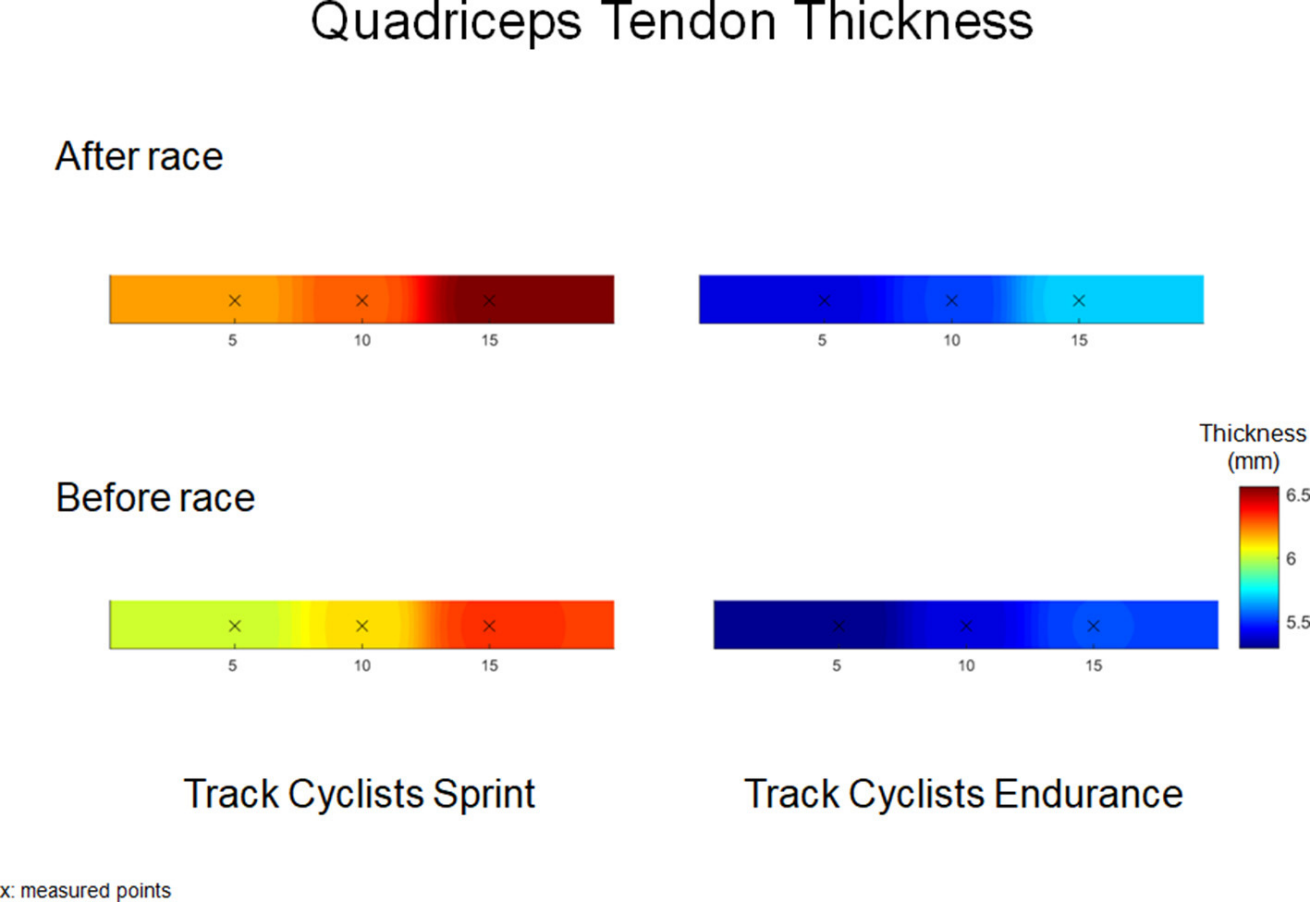
Quadriceps tendon thickness (mm) maps before and after 200 m flying start (Track Cyclists Sprint) and 4,000 m individual pursuit race (Track Cyclists Endurance).

**Figure 4:**
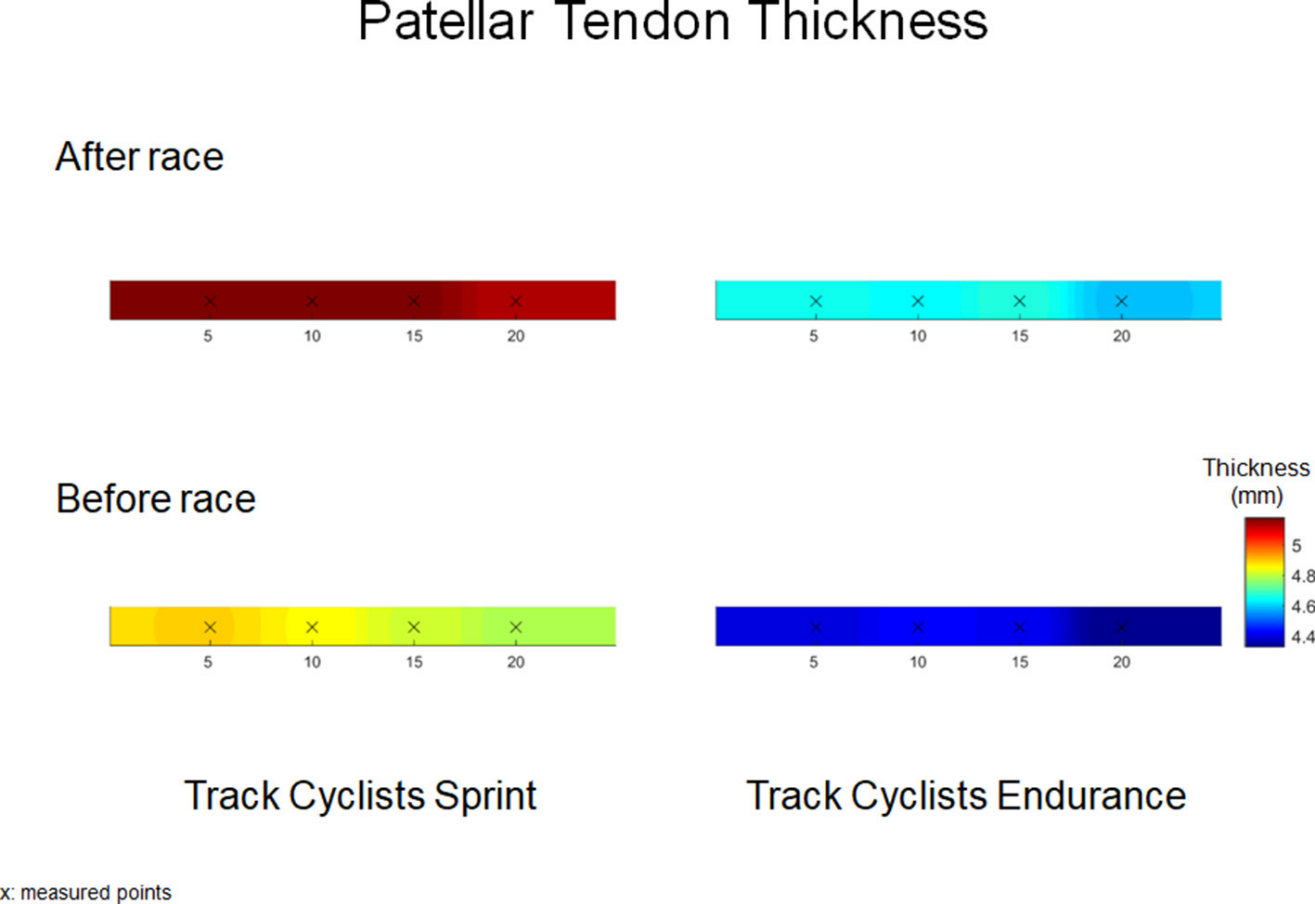
Patellar tendon thickness (mm) maps before and after 200 m flying start (Track Cyclists Sprint) and 4,000 m individual pursuit race (Track Cyclists Endurance).

Table 1 provides the mean (SD) and statistical analysis of QT_thick_ and PT_thick_ in sprint and endurance track cyclists. The two-way RM-ANOVAs revealed a statistically significant *Time* × *Location* interaction effect on averaged QT_thick_ [*F*_(2,36)_ = 31.9, *p* = 0.009, η^2^ = 0.69] and PT_thick_ [*F*_(2,38)_ = 99.2, *p* = 0.001, η^2^ = 0.70] in sprint track cyclists, as well as, QT_thick_ [*F*_(2,36)_ = 6.7, *p* = 0.02, η^2^ = 0.44] and PT_thick_ [*F*_(2,38)_ = 101.2, *p* = 0.02, η^2^ = 0.55] in endurance track cyclists. The analysis of within-group differences showed an increase in QT_thick_ [*F*_(1,28)_ = 4.7, *p* = 0.001, η^2^ = 0.71] and PT_thick_ [*F*_(1,32)_ = 7.3, *p* ≤ 0.001, η^2^ = 0.86] in sprint track cyclists after 200 m flying start. Similarly, an increase in QT_thick_ [*F*_(1,32)_ = 3.5, *p* = 0.01, η^2^ = 0.62] and PT_thick_ [*F*_(1,32)_ = 7.5, *p* ≤ 0.001, η^2^ = 0.86) was reported in endurance track cyclists after 4,000 m individual pursuit race. Moreover, the analysis revealed significant within-group differences compared to location placed at 5 mm (for 10 and 15 mm) in QT_thick_ in sprint (both *p* = 0.001) and endurance track cyclists (*p* = 0.001 and 0.01, respectively; Table 1, Figures 3, 4).

**Table 1:** Pre- and post-start QT_Thick_ and PT_Thick_ (mm) and PT_Stiff_ (N/m) in both groups using three-way analysis of variance.

**Variables**		**Sprint track cyclists**				**Endurance track cyclists**				
		**Pre**	**Post**	**Δ (post-pre)**	*P*-value^*^	**Pre**	**Post**	**Δ (post-pre)**	*p-*value^*^	*p-*value^**^
Qt_Thick_ (mm)	5	7.01 ± 0.09	7.19 ± 0.07	0.18	0.001	6.29 ± 0.04	6.40 ± 0.04	0.11	0.01	
	10	7.11 ± 0.06	7.28 ± 0.04^∧^	0.17	0.001	6.40 ± 0.06	6.51 ± 0.06∧	0.11	0.01	
	15	7.33 ± 0.06	7.56 ± 0.06^∧^	0.23	0.001	6.53 ± 0.05	6.70 ± 0.06∧	0.17	0.001	
Mean QT_thick_ (mm)		7.15 ± 0.16^#^	7.34 ± 0.19	0.19	0.001	6.41 ± 0.12^#^	6.54 ± 0.15	0.13	0.01	0.01
PT_thick_ (mm)	5	4.89 ± 0.05	5.18 ± 0.05	0.29	≤ 0.001	4.43 ± 0.11	4.66 ± 0.11	0.23	0.001	
	10	4.86 ± 0.08	5.17 ± 0.06	0.31	≤ 0.001	4.42 ± 0.11	4.64 ± 0.12	0.22	0.001	
	15	4.82 ± 0.09	5.18 ± 0.08	0.36	≤ 0.001	4.40 ± 0.15	4.67 ± 0.14	0.27	≤ 0.001	
	20	4.79 ± 0.09	5.13 ± 0.09	0.34	≤ 0.001	4.30 ± 0.11	4.59 ± 0.12	0.29	≤ 0.001	
Mean PT_thick_ (mm)		4.83 ± 0.08^#^	5.16 ± 0.09	0.33	≤ 0.001	4.39 ± 0.07^#^	4.64 ± 0.09	0.25	≤ 0.001	0.001
PT_Stiff_ (N/m)		527 ± 44^#^	731 ± 40	204	≤ 0.001	473 ± 35^#^	562 ± 32	89	0.001	≤ 0.001

Qt_Thick_, quadriceps tendon thickness; Pt_Thick_, patellar tendon thickness; PT_Stiff_, patellar tendon stiffness.

Significant differences

∧ within-group compared to location placed at 5 mm (p ≤ 0.05);

* within group differences between pre and post;

# between-group differences at baseline (p ≤ 0.05);

** *between-group differences post-pre race*.

At baseline, the one-way ANOVA showed significant higher averaged QT_thick_ [*F*_(1,15)_ = 98.1, *p* = 0.001, η^2^ = 0.82] and PT_thick_ [*F*_(1,14)_ = 73.5, *p* ≤ 0.001, η^2^ = 0.78] in sprinters compared with endurance track cyclists. Moreover, the post-pre race difference showed larger increase in QT_thick_ [*F*_(1,15)_ = 78.3, *p* = 0.01, η^2^ = 0.61] and PT_thick_ [*F*_(1,15)_ = 118.9, *p* ≤ 0.001, η^2^ = 0.75] in sprint compared with endurance track cyclists (Table 1).

### Myotonometry

The two-way RM-ANOVAs revealed a statistically significant *Time* × *Location* interaction effect on averaged QT_stiff_ [*F*_(5,59)_ = 133.5, *p* = 0.001, η^2^ = 0.72], PT_stiff_ [*F*_(5,59)_ = 81.5, *p* = 0.002, η^2^ = 0.72] and QM_stiff_ [*F*_(6,36)_ = 62.9, *p* = 0.005, η^2^ = 0.70] in sprint track cyclists, as well as, QT_stiff_ [*F*_(1,56)_ = 37.0, *p* = 0.01, η^2^ = 0.40], PT_stiff_ [*F*_(13, 60)_ = 86.7, *p* = 0.001, η^2^ = 0.62] and QM_stiff_ [*F*_(6,223)_ = 10.4, *p* = 0.01, η^2^ = 0.58] in endurance track cyclists. The analysis of within-group differences showed an increase in QT_stiff_ [*F*_(1,30)_ = 15.9, *p* ≤ 0.001, η^2^ = 0.82], PT_stiff_ [*F*_(1,32)_ = 22.3, *p* ≤ 0.001, η^2^ = 0.86], mean RF stiffness [*F*_(1,30)_ = 7.8, *p* = 0.04, η^2^ = 0.62], mean VL stiffness [*F*_(1,31)_ = 7.8, *p* ≤ 0.001, η^2^ = 0.83], QM_stiff_ [*F*_(1,32)_ = 7.5, *p* = 0.001, η^2^ = 0.80] in sprint track cyclists after 200 m flying start. Similarly, an increase in QT_stiff_ [*F*_(1,32)_ = 8.9, *p* = 0.001, η^2^ = 0.78], PT_stiff_ [*F*_(1,32)_ = 10.1, *p* = 0.001, η^2^ = 0.79] and mean RF stiffness [*F*_(1,31)_ = 8.4, *p* = 0.04, η^2^ = 0.64] was observed in endurance track cyclists after 4,000 m individual pursuit race (Tables 1, 2).

**Table 2:** Pre- and post-start QM_Stiff_ (N/m) at the seven measurement points using three-way analysis of variance.

**Reference points**	**Sprint track cyclists**				**Endurance track cyclists**				
	**Pre**	**Post**	**Δ (post-pre)**	*P*-Value^*^	**Pre**	**Post**	**Δ (post-pre)**	*p-*value^*^	*p-*value^**^
1	293 ± 34	339 ± 22	46	0.20	313 ± 31	342 ± 46	29	0.56	0.35
2	301 ± 21	379 ± 26	78	0.001	320 ± 32	380 ± 33	60	0.02	0.48
3	362 ± 62	396 ± 56	34	0.50	350 ± 48	368 ± 72	18	0.82	0.83
4	381 ± 73	464 ± 44	83^#^	0.001	378 ± 45	386 ± 52	11	0.83	0.02
5	349 ± 70	463 ± 43	114	≤ 0.001	379 ± 36	374 ± 46	−5	0.58	0.001
6	349 ± 45^#^	457 ± 58	108	≤ 0.001	299 ± 38^#^	378 ± 54	79	0.01	0.74
7	257 ± 32	347 ± 28	90	≤ 0.001	248 ± 32	299 ± 44	51	0.04	0.62
8	282 ± 50	416 ± 45	134	≤ 0.001	294 ± 32	357 ± 41	63	0.001	0.001
RF_mean_	292 ± 54	356 ± 45	64	0.04	314 ± 35	364 ± 46	50	0.04	0.76
VL_mean_	365 ± 64	463 ± 50	98^#^	≤ 0.001	348 ± 48	382 ± 52	34	0.62	0.05
QM_mean_	322 ± 44	408 ± 51	86	0.001	323 ± 45	367 ± 28	38	0.46	0.71

RF_mean_, mean stiffness of rectus femoris; VL_mean_, mean stiffness of vastus lateralis; QM_mean_, mean stiffness of quadriceps muscle.

Significant differences

* within group differences between pre and post;

# between-group differences at baseline (p ≤ 0.05);

** *between-group differences post-pre race*.

**Figure 5:**
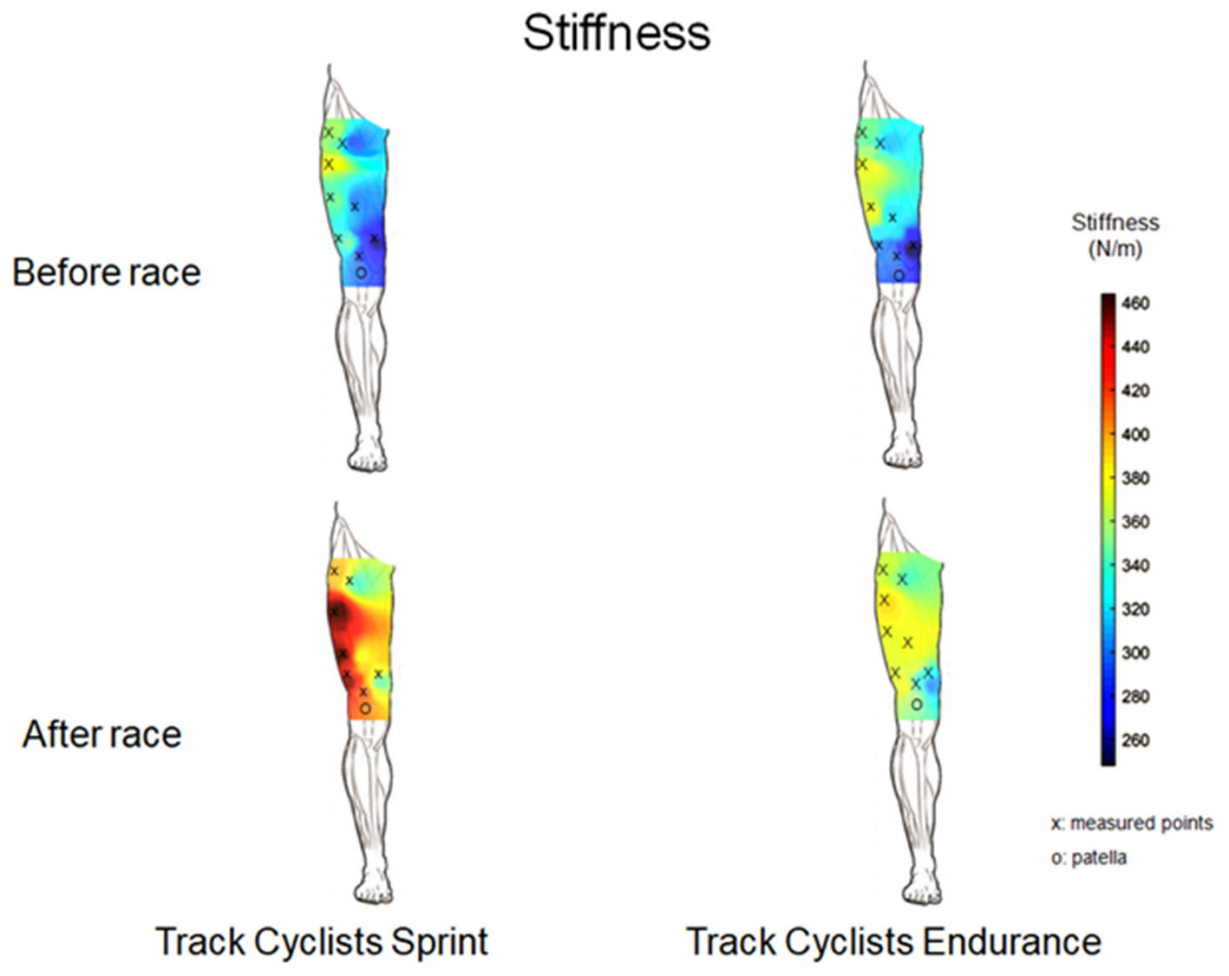
Muscle stiffness (N/m) maps from the quadriceps muscle and tendon before and after 200 m flying start (Track Cyclists Sprint) and 4,000 m individual pursuit race (Track Cyclists Endurance).

At baseline, the one-way ANOVA showed significantly higher PT_stiff_ [*F*_(1,14)_ = 6.9, *p* = 0.02, η^2^ = 0.55] in sprinters compared with endurance track cyclists. Moreover, the post-pre race difference showed higher QT_stiff_ [*F*_(1,15)_ = 79.2, *p* = 0.001, η^2^ = 0.72] and PT_stiff_ [*F*_(1,14)_ = 204.0, *p* ≤ 0.001, η^2^ = 0.85], as well as, mean VL stiffness [*F*_(1,89)_ = 113.2, *p* = 0.05, η^2^ = 0.64] in sprinters compared with endurance track cyclists (Tables 1, 2; Figure 5).

## Discussion

The current study showed an increase in QT and PT thickness and stiffness in both sprint and endurance cyclists after 200 and 4,000 m race. Sprint track cyclists were characterized by higher values of tendons thickness before the race than endurance track cyclists. The reported increase in both QT_thick_, PT_thick_, and QM_stiff_ in sprint track cyclists after 200 m flying start may indicate quadriceps muscle edema and hypervascularity. The results of our study are in line with the hypothesis, which predicts spatial changes of QM_stiff_ and acute alterations in tendons thickness after sprint and endurance races. The changes in QT_thick_ and PT_thick_ were assessed using diagnostic ultrasound imaging as well as the changes in QM_stiff_ and tendons stiffness was assessed using myotonometry at several locations depicting non uniform changes in both thickness and stiffness. Both ultrasound and myotonometry showed good to excellent relative reliability. These findings are interpreted in relation to the anthropometrics characteristics of sprint track cyclists (Van Der Zwaard et al., 2019) and competition characteristics (Craig and Norton, 2001) as well as with respect to the risk of getting injury.

### Physiological Perspective to Acute and Chronic Mechanisms Following Fatigue

*In vivo* studies have evaluated tendon's degenerative model described as acute laceration models induced damage include stress, fiber disorganization, inter-fiber tears, edema, and disrupted microfiber remodeling (Proske and Morgan, 2001; Nakama et al., 2005; Fung et al., 2010; Mccreesh et al., 2017). Furthermore, those changes in the structure of a tendon result in morphological and mechanical properties, including kinked fiber patterns causing torsion of fibers across and increased stiffness (Fung et al., 2010). We observed an increase of QT_thick_ and PT_thick_ in sprint and endurance track cyclists after races. Moreover, the difference between post-pre stiffness was larger in sprint compared with endurance track cyclists. Ditroilo et al. (2011) reported that stiffness increases linearly with increased tension, which is in relation with our previous study (Klich et al., 2020). We have shown an increase in QM tension and a decrease in elasticity, mostly as a result of glycogen depletion and increased production of lactate acid and hydrogen ions. Freedman et al. (2014) and Fung et al. (2010) have demonstrated the effect of tendon damage on its stiffness. The degenerative model assumes a decrease of stiffness in the second phase of post-exercise causing an increase in plastic deformation and fibers redistribute loads from damaged. Proske and Morgan (2001) have reported that increased stiffness is accompanied by micro swelling within the muscle fibers, however, we have not found any evidence. The higher post-pre differences in tendon thickness in sprint track cyclists might be related to alterations in tendon fluid since short-term efforts relapse glycosaminoglycan responsible for binding water (Mccreesh et al., 2017) causing direct influence on tendon stiffness and a result of higher tendon vascularity (Tsui et al., 2017).

The chronic model of overuse should be considered as a degenerative model including changes in connective tissues, e.g., collagen synthesis or fibroblast migration (Fredberg and Stengaard-Pedersen, 2008). Higher differences in sprinter's stiffness might be related to training specificity, e.g., short-term maximal efforts. Our previous study reported an increase in thigh muscle (especially VL) pain sensitivity after maximal anaerobic power training (Klich et al., 2018) and 200 m flying start (Klich et al., 2020). The training loads in endurance cyclists are mainly focused on aerobic capacity, and overloads does not seem to influence stiffness and thickness (Karamanidis and Arampatzis, 2006).

### Effect of 200 m Flying Start and 4,000 m Pursuit Race on QT_Thick_ and PT_Thick_

This study investigated the QT_thick_ and the PT_thick_ at three and four locations, respectively. Moreover, it is also the first study evaluating spatial changes in thickness and stiffness immediately after an elite event in track cyclists. Previous studies have also assessed the QT_thick_ and PT_thick_ at different locations from their attachments (Ozçakar et al., 2003; Pfirrmann et al., 2008; Giombini et al., 2013; Todd et al., 2015; Kizilkaya and Ecesoy, 2019). Several studies have examined both tendons thickness in elite sports (Pfirrmann et al., 2008; Giombini et al., 2013) measured at insertion, mid-length, and distal attachments. For instance, Giombini et al. (2013) reported tendon thickness 10 mm proximal (for QT) and 5 mm distal from the patella apex (for PT). Fisker et al. (2017) have evaluated PT_thick_ after CrossFit workout using similar ultrasound examination procedures to the one describe in Giombini et al. (2013). Ozçakar et al. (2003) evaluated the QT_thick_ in soccer players near to the insertion to the patella. Other studies reported the patient's QT_thick_ at 10 mm (Kizilkaya and Ecesoy) and 30 mm (Todd et al., 2015) proximal to the patella apex and the PT at 10 mm distal to the insertion. In our study, we investigated the QT_thick_ in locations placed 5–10–15 mm proximal and 5–10–15–20 mm distal to the apex of the patella. We observed significant differences in QT_thick_ and PT_thick_ between both groups at baseline, related with greater body mass of sprint track cyclists and influence of habitual loading during effort (Zhang et al., 2015; Mersmann et al., 2017). Our findings showed significant differences in spatial distribution of QT_thick_ in within-group compared to location placed at 5 mm in sprint and endurance track cyclists The results demonstrated significant highest QT_thick_ at locations placed 15 mm (difference in thickness ranged between 2 and 4%) in both groups, however in PT_thick_ were not found any statistical differences. Previous studies have assessed only a single measure 10 mm proximately from the apex of the patella (Ozçakar et al., 2003; Todd et al., 2015; Kizilkaya and Ecesoy, 2019); however, Fredberg et al. (2008) have reported that PT_thick_ was significantly larger in proximal areas as compared to distal locations.

After the sprint or endurance track, significant increases in both PT_thick_ and QT_thick_ were found. The percentage increase in QT_thick_ was 3% (sprint) and 2% (endurance), while PT_thick_ increased by 6 and 5%, respectively. The highest post-race increase in QT_thick_ was found at location placed 15 mm (Δ post-pre: 0.23 mm in the sprint and 0.17 mm in endurance track cyclists), while PT_thick_ at 15 mm (Δ post-pre: 0.36 mm) in sprinters and 20 mm (Δ post-pre: 0.29 mm) in endurance cyclists. Castro et al. (2019) have reported that the middle region of PT_Thick_ (~20 mm) was the easiest to define and had the highest intra-rater reliability. Such increases in the tendon thickness are most likely caused by including hypoechogenicity and vascularity (Visnes et al., 2015; Tsui et al., 2017). Visnes et al. (2015) reported that the hypoechoic areas are correlated with a risk for developing tendon tendinopathy. Previous studies have shown a training-induced adaptation of muscles and tendons by longtime training on morphological and mechanical in different athletes (Cook et al., 2004; Couppe et al., 2008; Giombini et al., 2013; Charcharis et al., 2019). However, our study presents an acute result of specific effort on tendon morphological and viscoelastic properties changes. Visnes et al. (2015) showed an increase of about 7–11% in the QT_thick_, without any changes in the PT_thick_. Furthermore, the authors observed a 50% thicker QT compared with PT among cyclists. Zhang et al. (2014) reported that morphological alterations in overloaded tendons (~23% difference in the PT_thick_). In our study, both tendons increased their thickness; however, a higher percentage increase was observed within the PT (~6–7%). In general, cycling training is based on short-term high-intensity exercises, strength training (sprint cyclists) (Klich et al., 2018), and aerobic training (endurance cyclists) (Craig and Norton, 2001; Faria et al., 2005). Thus, changes in morphological properties are mostly considered due to tendon adaptation (Visnes et al., 2015). Visnes et al. (2015) reported an increase of about 7–11% in the QT_thick_, without any changes in the PT_thick_. Magnusson and Kjaer (2003) observed that tendon hypertrophy in endurance athletes leads to a lower risk of stress across the tendon. The tendinopathy alterations may indicate intrinsic changes in the tendon due to vascularity, causing micro-inflammation (Craig and Norton, 2001; Seynnes et al., 2009). This finding has been reproduced by Fisker et al. (2017) who have also observed an impact of high-intensity loads on tendon thickening.

### Effect of 200 m Flying Start and 4,000 m Pursuit Race on QT_Stiff_, PT_Stiff_, and QM_Stiff_

Several studies assessed changes in viscoelastic properties due to training and fatigue (Seynnes et al., 2009; Mannarino et al., 2019; Chalchat et al., 2020), tendinopathy (Zhang et al., 2014), or muscular overload in elite athletes (Andonian et al., 2016; Young et al., 2018; Cristi-Sánchez et al., 2019). In our study, we observed significant differences in stiffness within groups and between-groups when comparing relative changes (difference post-pre race) in sprint and endurance track cyclists, in line with Epro et al. (2019). Previous studies have found an increase in the QT_stiff_ and the PT_stiff_ following isokinetic fatigue protocol (Chalchat et al., 2020), resistance training (Seynnes et al., 2009; Visnes et al., 2015; Mersmann et al., 2017; Mannarino et al., 2019) and prolonged exercise (Andonian et al., 2016). However, cross-sectional studies reported higher stiffness in different athletes compared with a healthily control group (Zhang et al., 2015; Young et al., 2018; Cristi-Sánchez et al., 2019). Taş et al. (2019) assessed stiffness of PT, QT, RF, and VM in soccer players compared with a control group. Soccer players had lower PT_stiff_ and QT_stiff_ as well as higher stiffness of RF compared with sedentary controls. Those authors have reported also higher PT_stiff_ then QT_stiff_ and similar stiffness of RF and VM in soccer players. Cristi-Sánchez et al. (2019) found higher PT_stiff_ in soccer players than inactive participants suggesting that the PT_stiff_ is related to the level of higher force transmission during muscle contraction, however Charcharis et al. (2019) reported higher thickness of VL and PT_stiff_ in athletes (different sports), as a result of training-induced adaptation. Mersmann et al. (2017) and Zhang et al. (2015) have observed higher PT_stiff_ in volleyball players, as compared with healthily volunteers, providing an evidence that PT is adapting to mechanical loading. Furthermore, their research showed following findings to the higher stiffness in athletes: (1) develops the performance of the muscle-tendon unit interaction [also supported by Arampatzis et al. (2020)] and (2) protects the muscle and tendons against overstrain. Visnes et al. (2015) and Mersmann et al. (2017) found that short-term training also result in higher PT_stiff_. Furthermore, those changes in the viscoelastic properties should be considered in relation to muscle strength (Epro et al., 2019) and increases in tendon's thickness (Klich et al., 2019). Seynnes et al. (2009) have suggested that training-induced changes may be related to tendon hypertrophy and alterations in collagen synthesis. While, Epro et al. (2019) has proposed that changes in tendon thickness may be the results of protective mechanisms.

The current study also assessed the spatial changes in the viscoelastic properties of QM_stiff_ after 200 m flying start or 4,000 m individual pursuit race. In our study, we observed an heterogeneous spatial distribution at baseline for QM_stiff_ in both groups, i.e., higher stiffness in TFL (point no. 3) and VL (point no. 4–6). After races, the spatial distribution remained similar with a shift toward higher stiffness. A higher increase in the QM_stiff_ in sprint track cyclists may indicate an adaptation to the specific-training loads (Cristi-Sánchez et al., 2019), especially due to mechanical energy transmission during cycling phases (Young et al., 2018). Moreover, Kordi et al. (2020) reported a positive relationships between quadriceps muscle volume and peak power output in sprint track cyclists. The alterations in QM_stiff_ after 200 m flying start, observed in our study could be considered by higher power-cadence and torque-cadence in sprinters, than in endurance track cyclists (Kordi et al., 2020). Furthermore, the differences in spatial distribution in QM_stiff_ between sprint and endurance track cyclists might be related with higher activity of QM during pedaling, especially activity of VL during the propulsion phase (Dorel et al., 2005), thus shows the highest activity during sprint exercises (Akima et al., 2005). Kordi et al. (2020) have not found any relationship between activity of VL and peak power output, and significant differences between muscle volume of VL in sprint and endurance track cyclists. In our study, differences in stiffness of VL between both groups could be explained by muscle fiber type composition of VL in sprint and endurance track cyclists. Higher QM_stiff_ may be related with greater proportion of type II muscle fibers in VL (Akima et al., 2005; Kordi et al., 2020). Additionally, higher stiffness in sprinters is also a result of muscle metabolic response, due to increases in lactate acid concentration and H^+^ ions (Visnes et al., 2015). It should be noted that sprint and endurance track cyclists have set their position on the bicycle differently. Therefore, this factor may influence those differences in the QM_stiff_. In a previous case report, we found increased stiffness in the QM during 200 m flying start and then sprint events, as a result of increased fatigue and higher maximal power output (Klich et al., 2020).

### Perspectives

Ultrasonography and myotonometry are frequently used to evaluate tendon and muscle tissues, in muscle strains, tendinopathies, tears, and overloading injuries. Field testing of potential tissue-overloading is very important when evaluating injury risk-factors and rehabilitation management. Furthermore, investigation of the quadriceps muscle among athletes may provide important critical clinical findings regarding morphological properties and, consequently injury mechanisms. Finally, measuring thickness at different locations and stiffness over QM enable to delineate spatial changes in the generate 3D graphical representations that can be used to monitor potential risk of injuries and optimize rehabilitation process.

### Limitations

Finally, we should recognize some potential limitations of the current study. First, we have investigated only the quadriceps femoris muscle. Future studies should also include hamstring and adductor muscles. Second, we could have investigated the cross-sectional area of the QT and the PT. However, this research protocol was prepared according to Ekizos et al. (2013) that consider measurements of cross-sectional area in PT not reliable. Third, we reported an acute effect in tendons; however, repeated measures after 12 and/or 24 h should be conducted in future studies to assess the recovery process following competitions. Future studies investigating QT_Thick_, PT_Thick_, and QM stiffness in sports why high knee-injury risks like handball are also warranted.

## Conclusions

This study reported for the first time changes in morphological and mechanical properties, represented by tendon thickness and stiffness. Moreover, our study showed spatial heterogeneity of tendons thickness and stiffness presented by 3D topographical maps after track cycling competition. Sprint track cyclists exhibited significantly higher post-pre differences in thickness and stiffness of QT and PT, as compared with endurance track cyclists. Moreover, the spatial differences in muscle stiffness and tendon thickness reported for both groups might be associated also with loading adaptation, and thus adaptation-based vascularity and hypertrophy processes. Higher stiffness in sprint compared with endurance track cyclists at baseline seems to highlight alterations in mechanical properties of the tendon and muscle that could lead to overuse injuries. The current study suggests that assessments using both ultrasonography and myotonometry provides crucial information about tendons and muscles properties and their acute adaptation to exercise.

## Data Availability Statement

The datasets generated for this study are available on request to the corresponding author.

## Ethics Statement

The studies involving human participants were reviewed and approved by Ethical Committee of the University Research Ethics Committee at the University School of Physical Education in Wrocław. The patients/participants provided their written informed consent to participate in this study.

## Author Contributions

SK, IK, and AKa planned this study. SK and IK collected data. SK, KF, PM, and AKl prepared the manuscript. PM and CF interpreted data and supervised the study. KF, PM, and AKa supervised method analysis. All authors read and approved the final version of the manuscript.

## Conflict of Interest

The authors declare that the research was conducted in the absence of any commercial or financial relationships that could be construed as a potential conflict of interest.
